# 13-Series resolvins mediate the leukocyte-platelet actions of atorvastatin and pravastatin in inflammatory arthritis

**DOI:** 10.1096/fj.201700268

**Published:** 2017-05-02

**Authors:** Mary E. Walker, Patricia R. Souza, Romain A. Colas, Jesmond Dalli

**Affiliations:** Lipid Mediator Unit, Centre for Biochemical Pharmacology, William Harvey Research Institute, Barts and the London School of Medicine, Queen Mary University of London, London, United Kingdom

**Keywords:** pharmacology, resolution, eicosanoids, ω-3, vascular inflammation

## Abstract

Rheumatoid arthritis is an inflammatory condition characterized by overzealous inflammation that leads to joint damage and is associated with an increased incidence of cardiovascular disease. Statins are frontline therapeutics for patients with cardiovascular disease and exert beneficial actions in rheumatoid arthritis. The mechanism that mediates the beneficial actions of statins in rheumatoid arthritis remains of interest. In the present study, we found that the administration of 2 clinically relevant statins—atorvastatin (0.2 mg/kg) or pravastatin (0.2 mg/kg)—to mice during inflammatory arthritis up-regulated systemic and tissue amounts of a novel family of proresolving mediators, termed 13-series resolvins (RvTs), and significantly reduced joint disease. Of note, administration of simvastatin (0.2 mg/kg) did not significantly up-regulate RvTs or reduce joint inflammation. We also found that atorvastatin and pravastatin each reduced systemic leukocyte activation, including platelet-monocyte aggregates (∼25–60%). These statins decreased neutrophil trafficking to the joint as well as joint monocyte and macrophage numbers. Atorvastatin and pravastatin produced significant reductions (∼30–50%) in expression of CD11b and major histocompatibility complex class II on both monocytes and monocyte-derived macrophages in joints. Administration of an inhibitor to cyclooxygenase-2, the initiating enzyme in the RvT pathway, reversed the protective actions of these statins on both joint and systemic inflammation. Together, these findings provide evidence for the role of RvTs in mediating the protective actions of atorvastatin and pravastatin in reducing local and vascular inflammation, and suggest that RvTs may be useful in measuring the anti-inflammatory actions of statins.—Walker, M. E., Souza, P. R., Colas, R. A., Dalli, J. 13-Series resolvins mediate the leukocyte-platelet actions of atorvastatin and pravastatin in inflammatory arthritis.

Cardiovascular disease (CVD) is the primary cause of global mortality, representing ∼30% of all deaths (World Health Organization 2012; *http://www.who.int/mediacentre/factsheets/fs317/en/*). In arthritides, cardiovascular complications are thought to arise from the chronic inflammatory state that characterizes these conditions and causes >50% of premature deaths in patients with arthritis (1, 2). Despite the high incidence of CVD, for many years research into mechanisms that are engaged by therapeutics in rheumatoid arthritis focused on joint protection, with limited evidence on their potential cardiovascular actions. Statins are a group of molecules that regulate circulating cholesterol levels by inhibiting the activity of 3-hydroxy-3-methyl-glutaryl-coenzyme A (HMGCA) reductase. These drugs are now widely used in the treatment of CVD as a result of the above effects and because of their limited adverse effects, and in 2005, their sales in the United States was estimated to be $19.7 billion (3). Recent evidence suggests that statins may also be useful in treating a number of inflammatory diseases, including rheumatoid arthritis, where atorvastatin (4–6), pravastatin (7), and simvastatin (8–11) are suggested to exert joint protective actions. It is now apparent that the protective actions of statins, at least in part, are independent of their ability to regulate HMGCA reductase activity (12); therefore, during the last few years, there has been significant interest in identifying the mechanisms that are responsible for these beneficial actions.

We recently found that, during bacterial infections, atorvastatin up-regulates the biosynthesis of a novel family of lipid mediators, termed 13-series resolvins (RvTs). This family is composed of 4 molecules, namely RvT1, RvT2, RvT3, and RvT4 (13). Here, atorvastatin *via* nitrosylation of endothelial cyclooxygenase-2 (COX-2) up-regulates the formation of 13R-HDPA (13*R*-hydroxy-7*Z*,10*Z*,13*R*,14*E*,16*Z*,19*Z*-docosapentaenoic acid), a key intermediate in the biosynthesis of RvT that is then donated to neutrophils where it is converted to bioactive molecules (13, 14). These mediators form part of a novel genus of endogenous host protective molecules, termed specialized proresolving mediators, that fine-tune the inflammatory response to promote its termination (12, 15–18). In ongoing infections, RvT down-regulates the formation of proinflammatory mediators, including eicosanoids and the pyroptotic cytokine IL-1β, as well as regulates leukocyte trafficking and bacterial clearance that promotes the resolution of infectious inflammation (13).

Given the potent actions exerted by RvT in the regulation of host responses and that atorvastatin promotes their formation during infectious inflammation, we questioned whether the joint protective actions of atorvastatin in inflammatory arthritis were mediated by these molecules. We then investigated whether atorvastatin dampened systemic inflammation in experimental arthritis by assessing its ability to regulate circulating leukocyte responses and the role of RvTs in mediating these actions. We also tested whether regulation of RvT was shared with pravastatin and simvastatin. Here, we found that atorvastatin and pravastatin differentially regulated RvT formation in plasma and joints of mice during experimental arthritis. Each of these statins also reduced joint and systemic leukocyte activation, systemic platelet activation, and joint disease. Of note, inhibition of RvT biosynthesis reversed both the local and systemic protective actions exerted by atorvastatin and pravastatin.

## MATERIALS AND METHODS

### Materials

Materials included liquid chromatography–grade water, methanol and acetic acid (Thermo Fisher Scientific, Waltham, MA, USA), Eclipse C18 Poroshell column (2.8 µm × 4.6 mm × 150 mm; Agilent Technologies, Santa Clara, CA, USA), and C18 solid-phase extraction columns (Biotage, Uppsala, Sweden); synthetic and authentic standards for liquid chromatography–tandem mass spectrometry quantitation and deuterium (d) internal standards (d_5_-RvD2, d_5_-lipoxin A_4_ (LXA_4_), d_4_-prostaglandin (PG) E_2_ and d_4_-leukotriene (LT) B_4_), which were purchased from Cambridge Bioscience (Cambridge, United Kingdom) or provided by Charles N. Serhan (Harvard Medical School, Boston, MA, USA); atorvastatin, pravastatin sodium salt, simvastatin sodium salt, and celecoxib (Cambridge Bioscience); Dulbecco’s PBS (DPBS; with and without calcium and magnesium), RPMI-1640, collagenase D, DNAseI, paraformaldehyde, Histopaque-1077, trisodium citrate, HBSS, penicillin-streptomycin, and dextran from *Leuconostoc* spp. (Mr 450,000–650,000), IL-1β, and TNF-α (Sigma-Aldrich, St. Louis, MO, USA); fetal bovine serum, CD11b-PE-Texas Red, CD62P-Brilliant Violet 650, and CountBright Absolute Counting Beads (Thermo Fisher Scientific); whole blood lysing reagents kit (Beckman Coulter, Brea, CA, USA); collagenase type II (Worthington Biochemical, Lakewood, NJ, USA); and anti-mouse IgG, CD64-PE, Ly6G-Alexa Fluor 700, Ly6C-Brilliant Violet 785, CD45-APC/Cy7, IA/IE-Brilliant Violet 650, CD11b-Alexa Fluor 488, Siglec F-APC, CD115-Brilliant Violet 711, CD43-Brilliant Violet 510, Ly6C-Alexa Fluor 488, CD11c-Brilliant Violet 785, CD115-Brilliant Violet 711, CD43-Alexa Fluor 647, and CD41-Brilliant Violet 510 (BioLegend, San Diego, CA, USA).

### Animals

Male C57BL/6 mice (11 wk old) were procured from Charles River Laboratories (Kent, United Kingdom). Experiments strictly adhered to United Kingdom Home Office regulations (Guidance on the Operation of Animals, Scientific Procedures Act) and Laboratory Animal Science Association Guidelines (Guiding Principles on Good Practice for Animal Welfare and Ethical Review Bodies). All animals were provided with standard laboratory diet and water *ad libitum* and kept on a 12-h light/dark cycle.

### Inflammatory arthritis

Male C57BL/6 mice (11 wk old) were administered K/BxN serum (100 µl, i.p.) on d 0 and 2 to initiate inflammatory arthritis (19). Mice were then given atorvastatin, pravastatin, simvastatin (0.2 mg/kg each), or vehicle (DPBS^−/−^ that contained 0.05% ethanol) *via* i.v. injection on d 3, 5, and 7. Clinical scores were monitored daily by using a 26-point arthritic scoring system. Swelling and redness of mouse ankles/wrists, pads, and digits were inspected daily as previously described (19). Blood and paws were collected at the indicated time intervals.

In select experiments, mice were given 10 mg/kg celecoxib 1 h before statin injections. Blood and paws were collected either on d 8 after arthritis onset or 2 h after statin injection on d 7.

### Flow cytometry

Whole blood was collected by using heparin-lined syringes *via* cardiac puncture. Cells were incubated with Fc-blocking IgG and fluorescent-labeled Abs (see Supplemental Methods for Ab details) for 45 min on ice. Cells were washed and incubated with 0.1% live/dead stain for 30 min on ice. Red blood cells were lysed and fixed using the whole blood lysing reagent Kit. Staining was then evaluated by using LSRFortessa cell analyzer (BD Biosciences, San Diego, CA, USA) and analyzed using FlowJo software (v.10; Tree Star, Ashland, OR, USA).

Paws were harvested and leukocytes were isolated as previously described (19). In brief, paws were incubated in RPMI-1640 that contained 0.5 µg/ml collagenase D and 40 µg/ml DNaseI at 37°C for 30 min with vigorous agitation. Isolated cells were passed through a 70-µM strainer and suspended in RPMI-1640 that contained 2 U/ml penicillin, 100 mg/ml streptomycin, and 10% fetal bovine serum, then centrifugated at 400 *g* for 10 min. Isolated cells were suspended in DPBS^−/−^ that contained 0.02% bovine serum albumin and 1% Fc-blocking IgG (v/v) and were incubated with 0.1% live/dead stain for 20 min on ice. Cells were washed using DPBS^−/−^ and incubated with fluorescent-labeled Abs (see Supplemental Methods for Ab details) for 45 min on ice. These were then washed and fixed using 1% paraformaldehyde. CountBright Absolute Counting Beads were used for leukocyte enumeration. Staining was evaluated using LSRFortessa cell analyzer and analyzed using FlowJo software.

### Human neutrophil–endothelial cell incubations

Umbilical cords were collected by the midwifery staff of the Maternity Unit, Royal London Hospital (protocol approved by East London and The City Health Authority Research Ethics Committee; 06/Q0605/40), and HUVECs were isolated as previously described (20). Cells were then incubated with IL-1β and TNF-α (10 ng/ml each, 16 h, 37°C, 5% CO_2_).

Neutrophils were isolated from the blood of healthy consenting donors in accordance with the Declaration of Helsinki and Queen Mary Research Ethics Committee (QMREC)–approved protocol (QMREC 2014:61). Blood was collected by using 3.2% sodium citrate as an anticoagulant and centrifuged (100 *g* for 20 min). Platelet-rich plasma was removed as previously described (13). Red blood cells were agglutinated by adding DPBS^−/−^ and 6% dextran, gently mixing and incubating at room temperature for 20 min. The resulting supernatant was layered over Histopaque-1077 and centrifuged (350 *g* for 30 mins). Red blood cells in the resulting pellet were lysed by using ice-cold water and transferring to HBSS. Cells were obtained *via* centrifugation at 250 *g* for 10 min.

Incubations were conducted as previously described (13). In brief, HUVECs (8.5 × 10^5^ cells/cm^2^) were incubated with IL-1β (10 ng/ml) and TNF-α (10 ng/ml) for 16 h, then with atorvastatin, pravastatin, or vehicle (DPBS that contained 0.05% ethanol) for 30 min at 37°C. Four million neutrophils were added to each well and cells were incubated for 60 min (37°C). Incubations were quenched with ice-cold methanol that contained deuterated internal standards and samples were taken for lipid mediator profiling.

### Lipid mediator profiling

Ice-cold methanol that contained 500 pg of each deuterated (d) internal standard—d_8_-5S- hydroxyeicosatetraenoic, d_4_-LT B_4_, d_5_-LXA_4_, d_4_-PGE_2_, and d_5_-RvD2—was added to samples. These represent the various chromatographic regions of identified lipid mediators to allow quantification and assessment of sample recovery. Lipid mediators were extracted and profiling conducted as previously described (13, 21, 22). In brief, lipid mediators were extracted by using solid-phase extraction techniques and ExtraHera (Biotage) carried out as previously described (22). Lipid mediators were identified and quantified by using liquid chromatography–tandem mass spectrometry–based lipid mediator profiling. Multiple reaction monitoring was carried out by using signature Q1 (parent ion) and Q3 (characteristic daughter ion) ion pairs for each molecule, which were acquired in negative ionization mode. Lipid mediators were identified in accordance with published criteria matching retention time and with a minimum of 6 diagnostic ions in the tandem mass spectrometry spectra (22).

### Histology

Paws were placed in 10% formaldehyde (v/v; in water that contained 0.65% Na_2_HPO_4_ and 0.4% NaH_2_PO_4_) for 48 h. These were then placed in 10% EDTA in DPBS for 2 wk. When decalcified, paws were embedded in wax as previously described (19). Four-micron sections were obtained and hematoxylin and eosin staining was carried out by the Barts Cancer Institute Pathology Core as previously described (19).

### Statistics

Results are presented as means ± sem. Differences between groups were tested by using GraphPad Prism 7 (GraphPad Software, La Jolla, CA, USA) and 1-way ANOVA with *post hoc* Dunnett’s, Sidak’s, or Tukey’s multiple comparisons test. Where appropriate, 1-sample Student’s *t* test compared with normalized vehicle or 2-way ANOVA were used. The criterion for statistical significance was *P* < 0.05.

## RESULTS

### Differential regulation of local and systemic RvT by atorvastatin, pravastatin, and simvastatin during inflammatory arthritis

**Figure 1.**
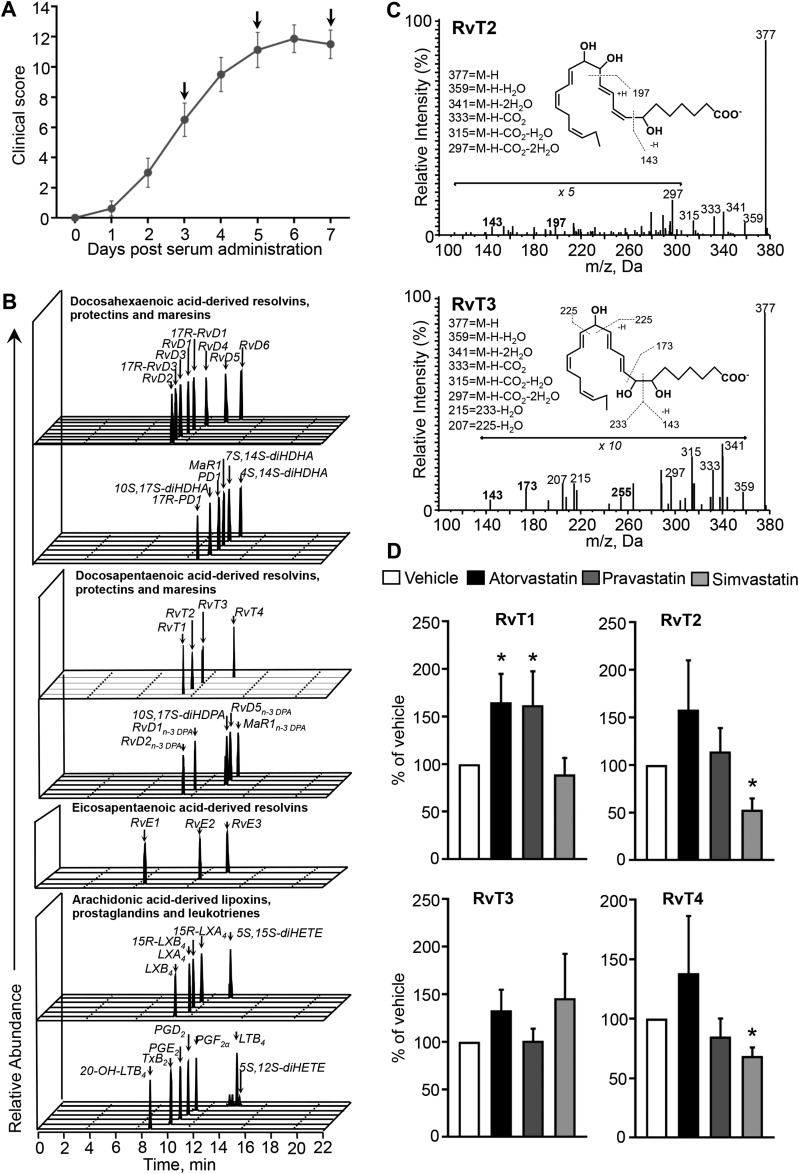
Increased RvTs in paws from mice that were administered atorvastatin and pravastatin during inflammatory arthritis. *A*) Arthritogenic K/BxN serum (100 μl, i.p.) was administered to mice to initiate disease, and disease progression was monitored daily using clinical scores. Arrows denote days when mice were given statins or vehicle (see Materials and Methods for details). *B*–*D*) Doses of 0.2 mg/kg atorvastatin, pravastatin, simvastatin, or vehicle (DPBS that contained 0.05% ethanol) were administered intravenously on d 3, 5, and 7 after disease onset. Paws were collected on d 8, and lipid mediators were identified and quantified by using lipid mediator profiling. *B*) Representative multiple reaction monitoring chromatograms of identified lipid mediators derived from docosahexaenoic acid, n-3 docosapentaenoic acid, eicosapentaenoic acid, and arachidonic acid. *C*) Tandem mass spectrometry spectra employed in the identification of RvT2 and RvT3. *D*) Percentage regulation of RvT1, RvT2, RvT3, and RvT4 compared with vehicle (*D*). Results are means ± sem; *n* = 9 for vehicle, 11 for atorvastatin, 11 for pravastatin, and 9 for simvastatin-treated mice from 4 independent experiments. *P < 0.05 *vs*. vehicle.

We first investigated whether atorvastatin regulated RvT formation during inflammatory arthritis and whether this action was unique to this statin or shared with other clinically relevant statins, namely pravastatin and simvastatin (7–11). To test this, we administered arthritogenic serum from K/BxN mice on d 0 and 2. This serum leads to an Fcγ-mediated immune response with a rapid onset and severe inflammatory arthritis (23). Mice were then administered atorvastatin (0.2 mg/kg), pravastatin (0.2 mg/kg), simvastatin (0.2 mg/kg), or vehicle in a therapeutic paradigm on d 3, 5, and 7 post–serum administration, at a time when clinical signs of disease were observed (**Fig. 1*A***). Plasma and paws were collected 24 h after the last statin dose, and lipid mediators were identified and quantified by using liquid chromatography–tandem mass spectrometry–based lipid mediator profiling. In paws from arthritic mice, we identified mediators from all 4 major bioactive metabolomes, including d-series resolvins and RvTs (Fig. 1*B*, *C*). These mediators were identified in accordance with published criteria, including matching retention times to authentic or synthetic standards (Fig. 1*B*) and at least 6 ions in the MS-MS (Fig. 1*C*) (21). Using multiple reaction monitoring, we determined the concentrations of mediators that were identified in these paws. Here, we found that in joints from mice that received atorvastatin there was a 43% increase in overall RvT amounts as a result of increased RvT1, RvT2, RvT3, and RvT4 compared with paws from vehicle-treated mice (Fig. 1*D* and Supplemental Table 1). Of note, the concentrations of these mediators were within their described bioactive ranges (13). Pravastatin also increased paw RvT by ∼20%, with increases in joint RvT1 and RvT2 concentrations, whereas simvastatin did not significantly increase joint RvT concentrations (Fig. 1*D* and Supplemental Table 1).

Assessment of plasma mediator levels demonstrated decreases in LT B_4_, PGD_2_, PGE_2_, PGF_2α_, and thromboxane B_2_ in mice that were administered either pravastatin or atorvastatin (Supplemental Table 2)_._ In these mice, we also found an increase in plasma levels of RvT concentrations, with RvT1 being increased by both atorvastatin and pravastatin, whereas RvT4 was only increased by atorvastatin (Supplemental Table 2)_._

Given that statins are rapidly cleared from circulation with a half-life of ∼14 h (24) and ∼3 h (25) for atorvastatin and pravastatin, respectively, we next investigated whether systemic regulation of RvT biosynthesis by these statins was more pronounced immediately after dosing. For this purpose, arthritis was initiated by using K/BxN serum and mice were administered atorvastatin and pravastatin as previously described. Blood was then collected 2 h after the last statin dose on d 7 and lipid mediators were identified and quantified by using lipid mediator profiling. In plasma from mice that were given atorvastatin, we found a significant increase (>200%) in RvTs, with increases in RvT1, RvT2, and RvT3 compared with vehicle-treated mice (Supplemental Fig. 1 and Supplemental Table 3). In these mice, we also found decreased circulating amounts of inflammation-initiating eicosanoids, including PGD_2_ and PGE_2_. Assessment of plasma lipid mediator profiles from mice that were administered pravastatin also demonstrated a marked increase (>100%) in peripheral blood RvTs, with RvT1 demonstrating the greatest increase compared with vehicle-treated mice (Supplemental Fig. 1 and Supplemental Table 3). In these mice, we also observed decreases in circulating PGD_2_ (∼46%) and PGE_2_ (∼29%). Together, these results demonstrate that atorvastatin and pravastatin increase both joint and plasma RvTs and decrease systemic inflammation during inflammatory arthritis.

Given that in the vasculature, RvT is produced during neutrophil endothelial interactions (13), we next questioned whether the increased RvT observed in murine systems by pravastatin was also translatable to humans. For this, we incubated human neutrophil–endothelial cell cocultures with pravastatin and assessed its ability to regulate RvTs. Here, we found that pravastatin dose-dependently up-regulated the concentrations of all 4 RvTs to a similar extent as that observed by atorvastatin (Supplemental Fig. 2).

### Atorvastatin and pravastatin reduce joint inflammation and protect against leukocyte-mediated tissue damage

**Figure 2.**
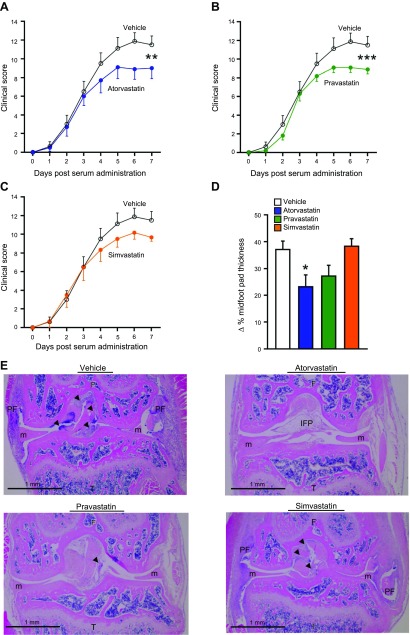
Atorvastatin and pravastatin reduced disease severity and protected joint architecture. Arthritogenic K/BxN serum was administered to mice on d 0 and 2. *A*–*C*) Disease progression was monitored by using a 26-point clinical score in mice given atorvastatin (0.2 mg/kg; *A*), pravastatin (0.2 mg/kg; *B*), simvastatin (0.2 mg/kg; *C*), or vehicle (DPBS that contained 0.05% ethanol) on d 3, 5, and 7. Results are means ± sem; *n* = 8 for vehicle, 10 for atorvastatin, 10 for pravastatin, and 6 for simvastatin-treated mice from 3 independent experiments. *D*) Maximum percentage increase in midfoot pad thickness. Results are means ± sem; *n* = 8 for vehicle, 10 for atorvastatin, 10 for pravastatin, and 6 for simvastatin-treated mice from 3 independent experiments. *E*) Representative hematoxylin and eosin–stained knee sections of mice collected on d 8 using EVOS FL imaging system. Original magnification, ×4. Results are representative of *n* = 8 for vehicle, 10 for atorvastatin, 10 for pravastatin, and 6 for simvastatin-treated mice from 3 independent experiments. Arrows denote leukocyte infiltration. F, femur; IFP, infrapatellar fat pad; m, meniscus; PF, pannus formation; T, tibia. *P < 0.05 *vs*. vehicle using 1-way ANOVA with *post hoc* Dunnett’s multiple comparisons test; **P < 0.01, ***P < 0.05 *vs*. vehicle using ordinary 2-way ANOVA.

We next investigated whether atorvastatin and pravastatin at doses that increased RvTs also reduced joint inflammation. Arthritis was initiated and mice were treated and disease progression monitored as described above. In mice that were administered vehicle, signs of disease were observed as early as d 2, and disease severity reached a maximum at d 6 with a score of 11.9 ± 0.9, after which disease activity plateaued to d 7 (**Fig. 2*A***). When mice were administered atorvastatin, disease progression was dampened as early as d 4 (1 d after treatment initiation), with disease scores reaching a maximum score of 9.1 ± 1.2 at d 5. This reduction in disease activity was sustained until d 7 (Fig. 2*A*). Similarly, when mice were administered pravastatin, disease activity at d 5 was found to be lower compared with mice that were administered vehicle alone, with a ∼23% reduction in disease activity that was maintained until d 7 (Fig. 2*B*). Administration of simvastatin at doses that were equal to those of atorvastatin and pravastatin did not significantly reduce disease activity (Fig. 2*C*). These findings were also reflected in the extent of paw swelling, where atorvastatin and pravastatin reduced joint swelling as measured by a decrease in midfoot pad thickness (Fig. 2*D*).

We next assessed whether atorvastatin, pravastatin, and simvastatin displayed joint protective actions. Hematoxylin and eosin–stained sections of knee joints from mice that were administered atorvastatin and pravastatin demonstrated reduced leukocyte infiltration, pannus formation, and joint damage compared with vehicle-treated mice, whereas these parameters were unaltered in mice that were administered simvastatin (Fig. 2*E*). Together, these findings demonstrate that atorvastatin and pravastatin are more potent than simvastatin at regulating local inflammation and protecting from leukocyte-mediated joint damage in inflammatory arthritis.

### Decreased leukocyte activation in joints and blood from arthritic mice by atorvastatin and pravastatin

**Figure 3.**
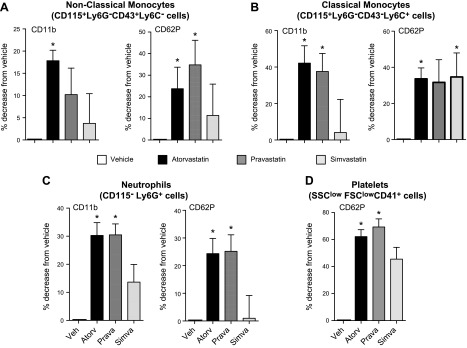
Differential regulation of circulating leukocyte and platelet activation by each of the statins in inflammatory arthritis. Serum-induced arthritis was initiated in mice and atorvastatin, pravastatin, simvastatin (0.2 mg/kg each), or vehicle (DPBS that contained 0.05% ethanol) were administered on d 3, 5, and 7. On d 8, blood was collected. Leukocyte subsets and activation were identified by using fluorescently labeled Abs and flow cytometry. Activation markers on nonclassic monocytes (*A*), classic monocytes (*B*), neutrophils (*C*), and platelets (*D*) were assessed as percentage decrease from vehicle. Results are means ± sem; *n* = 9 for vehicle, 11 for atorvastatin, 11 for pravastatin, and 9 for simvastatin-treated mice from 4 independent experiments. *P < 0.05 *vs*. vehicle using 1-way ANOVA with *post hoc* Dunnett’s multiple comparisons test.

To ascertain whether these statins regulated systemic inflammation in inflammatory arthritis, we assessed the levels of platelet–leukocyte aggregates in peripheral blood from arthritic mice, given the relationship of these heterotypic aggregates and cellular activation with CVD (26, 27). Using flow cytometry, we found that atorvastatin regulated the expression of CD11b on both nonclassic and classic monocytes as well as platelet–monocyte aggregates as measured by a decrease in CD62P (**Fig. 3*A*, *B***) and CD41 expression (*n* = 9 mice) on these monocyte subsets. Atorvastatin administration also regulated neutrophil and platelet responses, significantly reducing neutrophil CD11b expression, platelet-neutrophil aggregates (Fig. 3*C*), and decreasing platelet CD62P expression by ∼60% (Fig. 3*D*) compared with mice that were given vehicle alone. Similar findings were made with peripheral blood leukocytes and platelets from mice that were administered pravastatin, where there was a reduction in monocyte CD11b expression (∼10% for nonclassic and ∼40% for classic monocytes), platelet leukocyte aggregates (∼35% for both monocyte subsets and ∼25% for neutrophils), and platelet activation, with a reduction of ∼70% in CD62P expression (Fig. 3). Simvastatin did not significantly regulate leukocyte CD11b expression, whereas heterotypic aggregates that were formed by classic monocytes and platelets and platelet CD62P expression were reduced (Fig. 3). These results demonstrate that atorvastatin and pravastatin regulate systemic inflammation, dampening circulating monocyte, neutrophil, and platelet activation during inflammatory arthritis.

**Figure 4.**
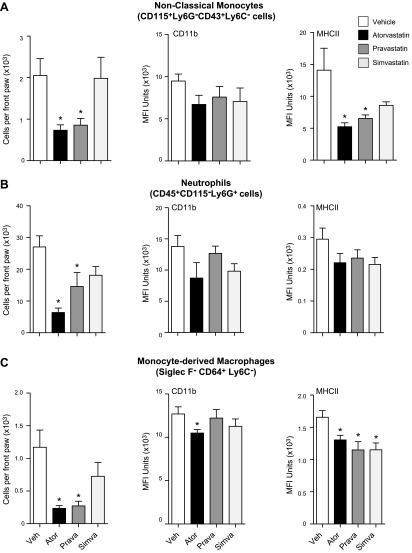
Reduction of monocyte, neutrophil, and macrophage activation, as well as trafficking to the joint, by atorvastatin and pravastatin in inflammatory arthritis. Serum-induced arthritis was initiated in mice and atorvastatin, pravastatin, simvastatin (0.2 mg/kg each), or vehicle (DPBS that contained 0.05% ethanol) were administered on d 3, 5, and 7. Front paws were collected on d 8 and digested to liberate infiltrating leukocytes. Leukocyte subsets were defined by using Abs against specific markers and flow cytometry. Trafficking and activation of nonclassic monocytes (*A*), neutrophils (*B*), and monocyte-derived macrophages (*C*) were assessed. Results are means ± sem; *n* = 9 for vehicle, 11 for atorvastatin, 11 for pravastatin, and 9 for simvastatin-treated mice from 4 independent experiments. *P < 0.05 *vs*. vehicle using 1-way ANOVA with *post hoc* Dunnett’s multiple comparisons test.

We next tested whether these actions also translated to a regulation of leukocyte trafficking and activation in the joint. We first assessed the trafficking of nonclassic monocytes to inflamed joints, given their role in disease onset and propagation of K/BxN serum–initiated inflammatory arthritis (28). Flow cytometric analysis of leukocytes that were isolated from joints of mice given atorvastatin demonstrated a significant reduction in the total numbers of nonclassic monocytes recruited to joints (>60%). There was also a reduction in CD11b and a significant reduction in major histocompatibility complex class II (MHCII) expression on these cells compared with mice that were administered vehicle alone (**Fig. 4*A***). We found that neutrophil trafficking was decreased in mice that received atorvastatin compared to vehicle-treated mice (Fig. 4*B*). In these mice, we found that statin administration down-regulated expression of neutrophil CD11b and MHCII, although this did not reach statistical significance (Fig. 4*B*). Assessment of macrophage trafficking to joints also demonstrated a significant reduction in the number of monocyte-derived macrophages as well as in the expression of activation markers CD11b and MHCII (Fig. 4*C*). Similar findings were also made with mice that were administered pravastatin with a reduction in joint monocyte, neutrophil, and macrophage numbers and activation profile (Fig. 4). Of note, although simvastatin regulated the expression of some activation markers on these cell subsets, it did not significantly reduce paw leukocyte numbers compared with mice given vehicle alone (Fig. 4). Together, these findings demonstrate that pravastatin and atorvastatin also regulate joint leukocyte trafficking and activation in inflammatory arthritis.

### COX-2 inhibition reverses protective actions of atorvastatin and pravastatin

**Figure 5.**
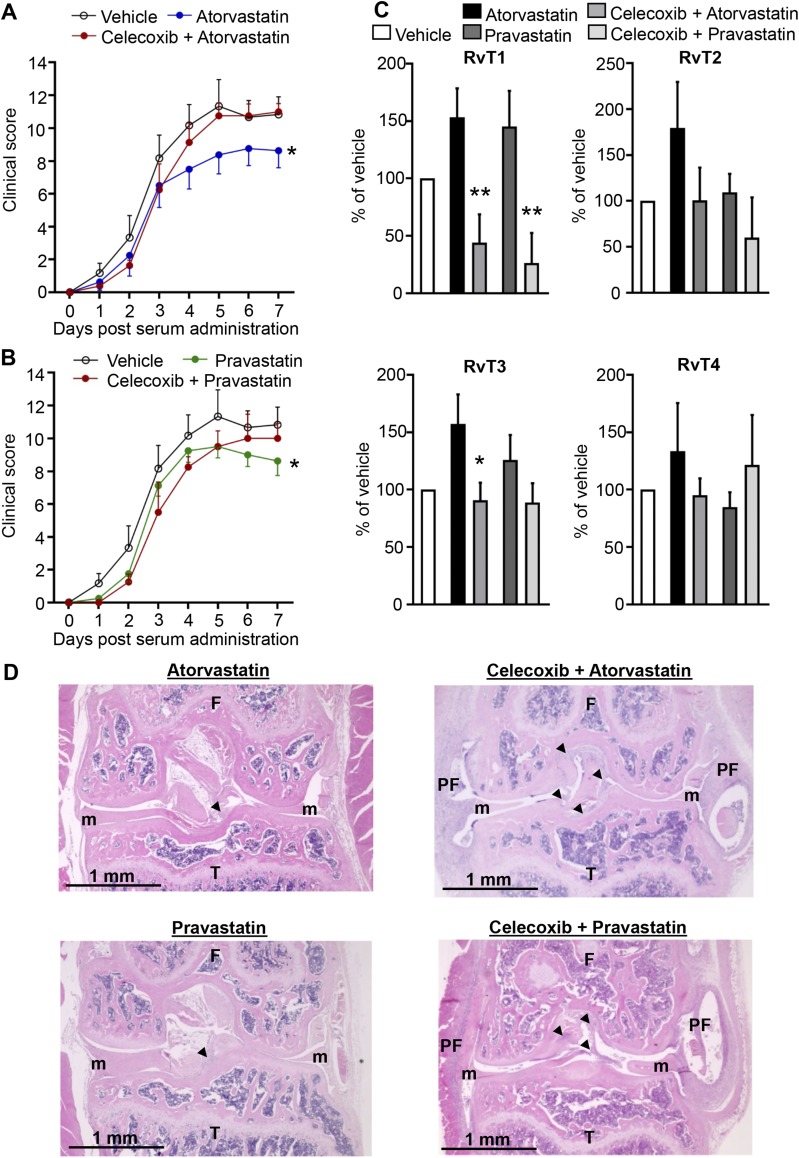
Inhibition of RvT production by celecoxib reverses the joint protective actions of atorvastatin and pravastatin. Inflammatory arthritis was initiated by using arthritogenic serum (see Materials and Methods for details). *A*, *B*) On d 3, 5, and 7, mice were administered celecoxib (10 mg/kg) or vehicle (DPBS that contained 0.05% ethanol) and after 1 h given atorvastatin (0.2 mg/kg; *A*), pravastatin (0.2 mg/kg; *B*), or vehicle (PBS that contained 0.05% ethanol). Disease activity was assessed daily. *C*) On d 8, paws were collected, and RvTs were identified and quantified by using liquid chromatography–tandem mass spectrometry–based lipid mediator profiling. *P < 0.05; **P < 0.01 *vs*. atorvastatin or pravastatin alone using 1-way ANOVA with *post hoc* Sidak’s multiple comparisons test. *D*) Representative hematoxylin and eosin–stained knee sections of mice collected on d 8 using EVOS FL imaging system. Original magnification, ×4. Results are means ± sem; *n* = 9 for vehicle, 11 for atorvastatin, 11 for pravastatin, 7 for celecoxib plus atorvastatin, and 6 for celecoxib plus pravastatin-treated mice per group from 2–3 independent experiments. *P < 0.05 *vs*. vehicle using ordinary 2-way ANOVA.

To assess the contribution of RvTs in the protective actions exerted by atorvastatin and pravastatin, we next investigated whether inhibition of COX-2—the initiating enzyme in the RvT pathway (13)—reversed the protective actions of pravastatin and atorvastatin. Clinical scores of mice that were administered celecoxib, a COX-2 selective inhibitor, immediately before atorvastatin were similar to those of mice that received vehicle alone and higher than those of mice that received atorvastatin (**Fig. 5*A***). Similarly, celecoxib also blunted the anti-inflammatory actions of pravastatin as measured by an increase in disease activity compared with mice that received the statin alone (Fig. 5*B*). This loss of the protective actions of pravastatin and atorvastatin in mice that were given celecoxib was also associated with an ∼60% reduction in joint RvT and RvT1 concentration that was >75% compared with mice that were given pravastatin or atorvastatin alone (Fig. 5*C*). In these mice, we also found a significant reversal of the joint protective actions of both statins where there was an increase in pannus formation and loss of joint architecture in mice that were given celecoxib compared with mice that were administered each statin alone (Fig. 5*D*).

**Figure 6.**
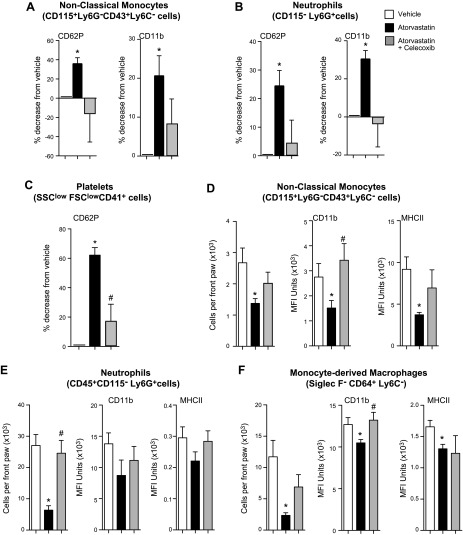
COX-2 inhibition reverses the protective actions of atorvastatin on both systemic and joint leukocytes. Serum-induced arthritis was initiated on d 3, 5, and 7 and mice were administered celecoxib (10 mg/kg) or vehicle (DPBS that contained 0.05% ethanol) and, after 1 h, given atorvastatin (0.2 mg/kg) or vehicle (DPBS that contained 0.05% ethanol). Blood was collected on d 8 and leukocyte subsets and activation were identified by using fluorescently labeled Abs and flow cytometry. *A*–*C*) Activation markers on circulating nonclassic monocytes (*A*), neutrophils (*B*), and platelets (*C*). Results are presented as percentage decrease from vehicle. *D*–*F*) Leukocytes were recovered from the inflamed paws (see Materials and Methods for details) on d 8. Trafficking and activation profile for nonclassic monocytes (*D*) neutrophils (*E*), and monocyte-derived macrophages (*F*) were assessed by using flow cytometry. Results are means ± sem; *n* = 9 for vehicle, 11 for atorvastatin, 11 for pravastatin, and 7 for celecoxib plus atorvastatin-treated mice from 2 independent experiments. *P < 0.05 *vs*. vehicle, ^#^*P* < 0.05 *vs*. atorvastatin using 1-way ANOVA with *post hoc* Dunnett’s multiple comparisons test.

We next investigated whether COX-2 inhibition also reversed the leukocyte-directed actions exerted by atorvastatin and pravastatin. Celecoxib administration blunted the protective actions of atorvastatin on circulating leukocytes and platelets, increasing blood platelet–monocyte and platelet–neutrophil aggregates as well as expression of CD11b on both leukocyte subsets (**Fig. 6*A*, *B***). Celecoxib administration also increased the expression of CD62P on circulating platelets compared with mice that received atorvastatin alone (Fig. 6*C*).

Inhibition of COX-2 reversed the actions of atorvastatin on leukocyte trafficking and activation in the joint, increasing the numbers of nonclassic monocytes (Fig. 6*D*), neutrophils (Fig. 6*E*), and monocyte-derived macrophages (Fig. 6*F*) that are recruited to inflamed joints. Expression of activation markers on these leukocytes was also increased after celecoxib inhibition compared with mice that were given atorvastatin alone (Fig. 6*D*–*F*). Similar findings were also made when we assessed systemic and joint leukocyte responses and trafficking in mice that were administered celecoxib together with pravastatin. Here, we found that COX-2 inhibition returned the activation profile of circulating leukocytes and platelets to that observed in mice receiving vehicle alone (Supplemental Fig. 3*A*–*C*). Similarly, leukocyte trafficking and activation in joints was increased to levels that were similar to those found in vehicle-treated mice (Supplemental Fig. 3*D–F*). Together, these findings demonstrate that inhibition of COX-2 reduced RvT production and abolished the joint and systemic protective actions of pravastatin and atorvastatin in inflammatory arthritis.

## DISCUSSION

Findings presented in this manuscript demonstrate that both atorvastatin and pravastatin increase RvT production in inflammatory arthritis. Up-regulation in tissue and blood concentrations of these specialized proresolving mediators was associated with a reduction in joint disease activity as well as joint leukocyte trafficking and activation. In addition, both atorvastatin and pravastatin decreased systemic inflammation, reducing platelet, monocyte, and neutrophil activation. The protective actions of these statins were reversed by COX-2 inhibition. Of note, simvastatin did not increase RvTs and displayed blunted actions in the regulation of joint disease and leukocyte responses. Together, these findings establish the rank order potency of atorvastatin, pravastatin, and simvastatin in regulating RvTs as well as the role of these molecules in mediating the protective actions of these statins.

Rheumatoid arthritis is characterized by an unabated inflammatory response that progressively leads to joint destruction and, ultimately, can be debilitating (29, 30). This persistent inflammatory response is also thought to be an underlying cause for the increased risk of developing CVD in patients with arthritis (1, 2). In this context, statins protect from CVD and recent studies suggest that they also reduce disease activity in patients with rheumatoid arthritis (4–11). It is evident that the pleiotropic actions of statins cannot be uniquely ascribed to inhibition of HMGCA reductase activity. Studies investigating potential mechanisms that may be engaged by statins found that atorvastatin (31) and lovastatin (32) up-regulate the formation of 15-epi-LXA_4_, which mediates their actions in cardiovascular and mucosal protection, respectively. In cardiac myocytes atrovastatin promotes 15-epi-LXA4 formation by increasing the phosphorylation of 5-lipoxygenase (31).

We recently demonstrated that, in the vasculature, atorvastatin up-regulates the formation of RvTs and that this pathway was responsible for mediating the protective actions of statins in bacterial infections (13). By using lipid mediator profiling, in the present study we found that this pathway was also regulated in the context of inflammatory arthritis by atorvastatin (Fig. 1 and Supplemental Figs. 1 and 2). We also found that the RvT pathway, at least in part, was responsible for the protective actions of this statin in controlling various aspects of inflammatory arthritis, including joint and vascular inflammation (Fig. 5). This regulation of the RvT pathway was shared with pravastatin that increased both joint and circulating RvT amounts, which suggests that these molecules mediate the systemic and local protective actions of these 2 statins. This is further supported by results obtained with both celecoxib and simvastatin, given that in either case, failure to up-regulate RvTs, in particular RvT1, was associated with increased disease activity (Figs. 5 and 6). Of note, in human neutrophil-endothelial cell incubations, simvastatin increased RvT levels (*n* = 4 healthy volunteers), although this was to a much lesser extent than atorvastatin and pravastatin. These results suggest that simvastatin is not as effective at promoting COX-2 S-nitrosylation as the other 2 statins. Furthermore, joint and plasma 15-epi-LXA_4_ were not significantly regulated in these mice, which underscores the role of RvTs in mediating the actions of these statins (Supplemental Tables 1–3).

Leukocytes, including monocytes, are implicated in the propagation of various aspects of CVD (26, 27). Studies investigating the role of different monocyte subsets in atherosclerosis, for example, found elevated classic and nonclassic monocytes in atherosclerotic plaques (33). Platelets play an important role in monocyte activation and transmigration. Monocyte–platelet aggregates lead to an enhanced ability of monocytes to interact with the vascular endothelium and increased monocyte transmigration (34). Platelet CD62P is critical in initiating platelet adhesion to circulating monocytes. This then leads to monocyte activation and an increased integrin expression, including CD11b, that mediates monocyte firm adhesion (34) and production of inflammatory cytokines and chemokines by monocytes, including TNF-α and C-C motif ligand 2 (35). These platelet–leukocyte aggregates are also a source of proinflammatory mediators, such as potent smooth muscle contractants LTC_4_ and LTD_4_ (36). In the present study, we found that both atorvastatin and pravastatin reduced circulating monocyte and platelet activation as well as monocyte–platelet aggregates, an action that was reversed after COX-2 inhibition (Fig. 6 and Supplemental Fig. 3). These findings support the role of RvTs in mediating the protective actions of these 2 statins in regulating systemic inflammation and processes that are associated with the development of CVD.

In rheumatoid arthritis, unabated leukocyte recruitment and activation leads to a perpetuation of joint inflammation and joint destruction. Among the leukocytes that are recruited to the joint, nonclassic monocytes were recently found to be essential in initiating joint inflammation in murine arthritis (28), whereas neutrophils play an important role in perpetuating joint damage *via* release of proteinases and production of extracellular traps (37). The inflammatory milieu found in the joint is also thought to be responsible for the differentiation of recruited monocytes to inflammatory macrophages that amplify joint inflammation by producing inflammatory mediators, including cytokines and chemokines, and activating the adaptive immune system (38). Results from the present study demonstrate that atorvastatin and pravastatin also regulate joint leukocyte recruitment and activation, reducing nonclassic monocyte, neutrophil, and macrophage numbers as well as the expression of activation markers. These actions were mediated by RvTs, as inhibiting their production reversed the protective actions of both statins and lead to an increase in joint inflammation (Figs. 5 and 6). Regulation of both systemic and local leukocyte responses is also in line with the biologic actions of RvTs. Indeed, these mediators potently regulate human leukocyte responses at concentrations identified in plasma and tissues in the present study (13). This is also in line with the biologic actions of other proresolving mediators that stereospecifically regulate monocyte, macrophage, and neutrophil activation (15–17, 29, 39, 40).

Taken together, these results establish the rank order potencies of atorvastatin, pravastatin, and simvastatin at regulating RvTs. Joint and systemic increases in these proresolving mediators also correlated with the ability of each of these statins to dampen various aspects of local and systemic inflammation, including edema, leukocyte, and platelet activation. Thus, these results establish a novel mechanism of action for atorvastatin and pravastatin in regulating inflammation in arthritis. In addition, they also provide potential novel functional biomarkers for measuring the efficacy of statins in controlling local and vascular inflammation in patients with rheumatoid arthritis.

## Supplementary Material

Supplemental Data

## Abbreviations

COX-2: cyclooxygenase-2
CVD: cardiovascular disease
DPBS: Dulbecco’s PBS
HMGCA: 3-hydroxy-3-methyl-glutaryl-coenzyme A
LT: leukotriene
MHCII: major histocompatibility complex class II
LXA_4_: lipoxin A_4_
PG: prostaglandin
RvT: 13-series resolvin
