# Supplementary material for: 13-Series resolvins mediate the leukocyte-platelet actions of atorvastatin and pravastatin in inflammatory arthritis
Source: FASEB J. 2017 May 2;31(8):3636–48. doi: 10.1096/fj.201700268 (PMC5503705; doi:10.1096/fj.201700268)
Supplement: Supplemental Data [file supp_31_8_3636__index.html]

13-Series resolvins mediate the leukocyte-platelet actions of atorvastatin and pravastatin in inflammatory arthritis — 13-Series resolvins mediate the leukocyte-platelet actions of atorvastatin and pravastatin in inflammatory arthritis — Supplemental Data 

# 13-Series resolvins mediate the leukocyte-platelet actions of atorvastatin and pravastatin in inflammatory arthritis

## Supplemental Data

- Supplemental Data
