# Supplementary material for: 13-Series resolvins mediate the leukocyte-platelet actions of atorvastatin and pravastatin in inflammatory arthritis
Source: FASEB J. 2017 May 2;31(8):3636–48. doi: 10.1096/fj.201700268 (PMC5503705; doi:10.1096/fj.201700268)
Supplement: Supplemental Data [file supp_fj.201700268_Supplemental_Data.pdf]

Supplemental Table 1: Joint LM-SPM profiles during inflammatory arthritis.

**DHA Bioactive Metabolome**	Q1	Q3	Vehicle (pg/paw)	Atorvastatin (pg/paw)	Pravastatin (pg/paw)	Simvastatin (pg/paw)
RvD1	375	233	1.4 ± 0.4	2.4 ± 0.6	1.7 ± 0.7	2.5 ± 0.9
RvD2	375	141	1.0 ± 0.3	1.4 ± 0.4	1.1 ± 0.3	0.9 ± 0.5
RvD3	375	147	0.3 ± 0.1	0.2 ± 0.1	0.2 ± 0.1	0.2 ± 0.1
RvD4	375	101	2.3 ± 0.4	**1.4 ± 0.2**	1.7 ± 0.3	**1.2 ± 0.3**
RvD5	359	199	20.2 ± 5.9	27.3 ± 7.8	22.6 ± 6.9	16.6 ± 8.0
RvD6	359	101	2.7 ± 0.7	2.7 ± 0.6	2.4 ± 0.5	2.0 ± 0.7
17*R*-RvD1	375	233	1.2 ± 0.5	0.7 ± 0.3	1.0 ± 0.3	0.9 ± 0.3
17*R*-RvD3	375	147	0.1 ± 0.0	0.1 ± 0.1	0.1 ± 0.0	0.1 ± 0.0
PD1	359	153	41.2 ± 17.5	59.1 ± 37.1	29.8 ± 9.3	39.2 ± 23.0
10S,17S-diHDHA	359	153	420.7 ± 152.2	506.6 ± 151.0	412.7 ± 114.0	406.8 ± 203.1
17*R*-PD1	359	153	34.3 ± 10.3	45.0 ± 11.9	43.1 ± 14.2	39.0 ± 21.5
MaR1	359	221	7.1 ± 1.4	12.6 ± 3.5	9.9 ± 2.9	8.6 ± 4.0
7S,14S-diHDHA	351	221	32.7 ± 0.6	7.1 ± 1.8	3.4 ± 1.3	2.1 ± 0.8
**n-3 DPA Bioactive Metabolome**						
RvT1	377	193	2.7 ± 0.4	**4.5 ± 0.7**	**4.3 ± 0.6**	2.6 ± 0.7
RvT2	377	215	0.5 ± 0.1	0.8 ± 0.2	0.6 ± 0.2	**0.3 ± 0.1**
RvT3	377	143	0.9 ± 0.3	1.1 ± 0.2	1.0 ± 0.2	1.3 ± 0.4
RvT4	361	193	4.3 ± 0.6	5.8 ± 1.0	3.5 ± 0.6	**3.0 ± 0.3**
$RvD1_{n\text{-}3\ DPA}$	377	143	1.4 ± 0.8	0.9 ± 0.3	0.9 ± 0.3	0.1 ± 0.1
$RvD2_{n\text{-}3\ DPA}$	377	233	1.0 ± 0.6	0.6 ± 0.2	0.7 ± 0.3	0.7 ± 0.3
$RvD5_{n\text{-}3\ DPA}$	361	199	2.6 ± 0.6	2.7 ± 1.1	2.5 ± 0.8	1.3 ± 0.5
$PD1_{n\text{-}3\ DPA}$	361	155	0.3 ± 0.1	0.9 ± 0.4	0.5 ± 0.2	0.2 ± 0.1
$MaR1_{n\text{-}3\ DPA}$	361	223	1.0 ± 0.3	1.2 ± 0.3	1.0 ± 0.3	0.5 ± 0.3
**EPA Bioactive Metabolome**						
RvE1	349	195	0.1 ± 0.1	0.3 ± 0.2	0.4 ± 0.2	-
RvE2	333	199	5.7 ± 3.5	3.6 ± 2.0	4.1 ± 2.6	5.9 ± 2.8
RvE3	333	201	0.6 ± 0.3	0.7 ± 0.4	0.3 ± 0.1	0.7 ± 0.4
**AA Bioactive Metabolome**						
LXA_4	351	217	0.2 ± 0.1	0.2 ± 0.1	0.2 ± 0.1	0.1 ± 0.1
LXB_4	351	221	13.3 ± 7.3	31.3 ± 21.3	22.4 ± 16.0	-
5S,15S-diHETE	335	115	16.5 ± 3.1	44.1 ± 15.9	27.9 ± 6.1	15.3 ± 5.3
15-epi-LXA_4	351	217	25.4 ± 10.5	17.1 ± 4.6	17.7 ± 3.9	9.2 ± 2.1
15-epi-LXB_4	351	221	2.0 ± 0.6	**0.8 ± 0.3**	1.5 ± 0.5	**0.6 ± 0.2**
LTB_4	335	195	27.4 ± 5.7	24.1 ± 11.4	32.0 ± 10.2	26.8 ± 9.7
PGD_2	351	189	3618.4 ± 1160.3	3794.1 ± 863.0	3352.3 ± 730.4	4640.6 ± 1203.9
PGE_2	351	189	3225.0 ± 849.3	3592.0 ± 654.4	3156.0 ± 649.3	4059.9 ± 961.0
$PGF_{2\alpha}$	353	193	1124.0 ± 433.0	1106.7 ± 275.6	690.1 ± 130.0	1452.1 ± 446.2
TxB_2	369	169	548.4 ± 145.5	725.5 ± 172.2	430.1 ± 89.7	643.6 ± 141.8

Paws were collected from mice 8 days after K/BxN-induced arthritis and giving atorvastatin, pravastatin, simvastatin (0.2mg/Kg) or vehicle (DPBS containing 0.05% ethanol). Lipid mediators were quantified using multiple reaction monitoring of the parent ion (Q1) and characteristic daughter ion (Q3). Results are expressed as mean ± s.e.m.; *n* = 9-10 mice per group, 3 independent experiments. - = below limits of detection; limits of detection ~0.1 pg. Values denoted in bold are $P < 0.05$ *vs.* vehicle .

Supplemental Table 2: Day 8 plasma LM-SPM during inflammatory arthritis.

**DHA Bioactive metabolome**	Q1	Q3	Vehicle (pg/mL)	Atorvastatin (pg/mL)	Pravastatin (pg/mL)	Simvastatin (pg/mL)
RvD1	375	233	6.9 ± 4.6	10.8 ± 7.8	11.5 ± 5.7	5.4 ± 1.1
RvD2	375	141	7.7 ± 3.9	11.3 ± 8.9	13.7 ± 7.9	1.7 ± 0.8
RvD3	375	147	0.4 ± 0.2	0.8 ± 0.5	0.6 ± 0.3	0.4 ± 0.1
RvD4	375	101	15.7 ± 3.8	21.8 ± 11.5	20.7 ± 8.2	8.0 ± 2.5
RvD5	359	199	11.4 ± 5.6	6.0 ± 1.9	8.3 ± 1.9	10.6 ± 3.9
RvD6	359	101	6.4 ± 1.9	4.8 ± 1.4	4.0 ± 1.3	2.7 ± 0.7
17*R*-RvD1	375	233	3.9 ± 1.1	3.1 ± 1.4	3.9 ± 1.8	1.7 ± 0.6
17*R*-RvD3	375	147	0.3 ± 0.2	0.2 ± 0.1	0.2 ± 0.1	0.4 ± 0.1
PD1	359	153	4.9 ± 1.9	27.5 ± 15.8	24.2 ± 16.9	8.1 ± 3.1
10*S*,17*S*-diHDHA	359	153	95.3 ± 41.1	111.5 ± 40.2	152.7 ± 37.5	225.5 ± 100.7
17*R*-PD1	359	153	5.8 ± 2.2	6.9 ± 2.5	10.0 ± 3.5	10.3 ± 3.7
MaR1	359	221	39.8 ± 14.7	49.5 ± 17.1	31.6 ± 11.1	15.8 ± 3.4
7*S*,14*S*-diHDHA	351	221	10.8 ± 3.7	11.4 ± 3.8	11.1 ± 4.6	7.5 ± 4.4
**n-3 DPA Bioactive Metabolome**						
RvT1	377	193	3.8 ± 1.4	4.8 ± 1.5	8.4 ± 3.1	1.8 ± 0.9
RvT2	377	215	2.3 ± 1.2	3.0 ± 1.3	1.4 ± 0.4	3.3 ± 1.9
RvT3	377	143	3.6 ± 1.5	1.8 ± 0.7	3.0 ± 1.4	3.6 ± 2.0
RvT4	361	193	3.2 ± 0.8	5.8 ± 2.9	3.3 ± 1.1	**0.7 ± 0.4**
RvD1$_{n-3\ DPA}$	377	143	5.0 ± 1.8	**2.9 ± 1.1**	5.2 ± 2.6	**0.8 ± 0.3**
RvD2$_{n-3\ DPA}$	377	233	9.0 ± 4.5	6.6 ± 4.4	11.0 ± 5.1	6.1 ± 3.9
RvD5$_{n-3\ DPA}$	361	199	2.5 ± 1.2	1.8 ± 0.6	2.6 ± 1.0	1.0 ± 0.3
PD1$_{n-3\ DPA}$	361	155	3.6 ± 1.6	3.1 ± 1.4	3.7 ± 1.7	0.7 ± 0.3
MaR1$_{n-3\ DPA}$	361	223	5.4 ± 1.8	6.0 ± 2.1	3.0 ± 1.1	1.8 ± 1.0
**EPA Bioactive Metabolome**						
RvE1	349	195	1.4 ± 0.8	4.4 ± 2.1	1.2 ± 0.7	**7.1 ± 3.2**
RvE2	333	199	2.8 ± 1.1	4.6 ± 1.5	2.5 ± 1.4	1.4 ± 0.5
RvE3	333	201	5.3 ± 2.4	2.5 ± 0.6	1.3 ± 0.4	2.5 ± 1.2
**AA Bioactive Metabolome**						
LXA$_4$	351	217	1.5 ± 1.0	3.2 ± 2.1	2.4 ± 2.0	0.1 ± 0.1
LXB$_4$	351	221	48.2 ± 31.8	57.0 ± 32.9	27.3 ± 18.1	35.6 ± 35.3
5*S*,15*S*-diHETE	335	115	21.9 ± 7.9	41.2 ± 19.8	38.6 ± 9.9	16.4 ± 4.4
15-epi-LXA$_4$	351	217	115.6 ± 32.6	149.9 ± 59.5	132.5 ± 47.1	42.6 ± 16.8
15-epi-LXB$_4$	351	221	18.9 ± 9.9	7.8 ± 3.3	8.5 ± 4.1	5.9 ± 3.3
LTB$_4$	335	195	182.4 ± 112.1	78.9 ± 28.0	110.1 ± 24.5	137.9 ± 79.5
PGD$_2$	351	189	832.0 ± 655.2	218.2 ± 131.9	465.4 ± 187.1	405.9 ± 250.9
PGE$_2$	351	189	1741.3 ± 1461.5	564.1 ± 448.5	610.0 ± 388.9	1080.3 ± 938.4
PGF$_{2\alpha}$	353	193	883.9 ± 779.8	224.2 ± 144.5	263.8 ± 124.1	476.2 ± 340.8
TxB$_2$	369	169	3017.3 ± 1975.5	1218.2 ± 408.1	1651.8 ± 721.0	3765.1 ± 1259.1

Plasma was collected from mice giving atorvastatin, pravastatin, simvastatin (0.2mg/Kg) or vehicle (DPBS containing 0.05% ethanol) 8 days after disease onset. Lipid mediators were identified in accordance with published criteria and quantified using multiple reaction monitoring of the parent ion (Q1) and characteristic daughter ion (Q3). Results are expressed as mean ± s.e.m.; *n* = 9-10 mice per group from 3 independent experiments. Values denoted in bold are $P < 0.05$ *vs.* vehicle.

Supplemental Table 3: Plasma lipid mediators 2 hours after statin administration.

**DHA Bioactive Metabolome**	Q1	Q3	Vehicle (pg/mL)	Atorvastatin (pg/mL)	Pravastatin (pg/mL)
RvD1	375	233	0.5 ± 0.1	0.7 ± 0.3	0.5 ± 0.1
RvD2	375	141	1.7 ± 0.6	1.3 ± 0.4	1.1 ± 0.6
RvD3	375	147	0.2 ± 0.0	0.2 ± 0.0	0.1 ± 0.0
RvD4	375	101	2.3 ± 0.5	3.1 ± 0.8	2.5 ± 0.3
RvD5	359	199	0.4 ± 0.1	0.5 ± 0.1	0.9 ± 0.6
RvD6	359	101	0.3 ± 0.1	0.3 ± 0.0	0.4 ± 0.1
17*R*-RvD1	375	233	0.5 ± 0.2	0.3 ± 0.0	0.6 ± 0.2
17*R*-RvD3	375	147	0.1 ± 0.0	0.1 ± 0.1	0.1 ± 0.0
PD1	359	153	0.6 ± 0.5	0.2 ± 0.1	0.3 ± 0.1
10*S*,17*S*-diHDHA	359	153	29.8 ± 16.6	6.5 ± 1.4	5.7 ± 1.2
17*R*-PD1	359	153	2.2 ± 1.1	1.2 ± 0.8	0.3 ± 0.1
MaR1	359	221	3.8 ± 0.9	5.9 ± 1.2	4.2 ± 1.2
7S,14S-diHDHA	351	221	13.4 ± 3.4	29.4 ± 11.6	11.5 ± 3.3
**n-3 DPA Bioactive Metabolome**					
RvT1	377	193	0.2 ± 0.1	**1.0 ± 0.2**	**0.9 ± 0.2**
RvT2	377	215	0.2 ± 0.1	**0.7 ± 0.1**	0.3 ± 0.1
RvT3	377	143	0.1 ± 0.0	0.5 ± 0.2	0.2 ± 0.0
RvT4	361	193	0.2 ± 0.0	0.2 ± 0.0	0.1 ± 0.0
RvD1$_{n\text{-}3\ DPA}$	377	143	0.9 ± 0.3	3.2 ± 2.4	0.8 ± 0.3
RvD2$_{n\text{-}3\ DPA}$	377	233	1.9 ± 0.4	2.5 ± 0.9	1.3 ± 0.3
RvD5$_{n\text{-}3\ DPA}$	361	199	0.2 ± 0.0	0.2 ± 0.0	0.2 ± 0.1
PD1$_{n\text{-}3\ DPA}$	361	155	0.3 ± 0.0	0.4 ± 0.1	0.4 ± 0.2
MaR1$_{n\text{-}3\ DPA}$	361	223	0.5 ± 0.1	0.6 ± 0.1	0.4 ± 0.1
**EPA Bioactive Metabolome**					
RvE1	349	195	0.3 ± 0.1	0.3 ± 0.1	0.3 ± 0.0
RvE2	333	199	0.7 ± 0.2	0.5 ± 0.2	1.0 ± 0.4
RvE3	333	201	0.6 ± 0.1	**1.0 ± 0.1**	0.9 ± 0.1
**AA Bioactive Metabolome**					
LXA_4	351	217	1.1 ± 0.4	0.9 ± 0.4	0.4 ± 0.2
LXB_4	351	221	0.7 ± 0.1	1.1 ± 0.5	1.0 ± 0.5
5S,15S-diHETE	335	115	1.7 ± 0.4	0.8 ± 0.2	0.8 ± 0.4
15-epi-LXA_4	351	217	0.2 ± 0.1	0.5 ± 0.2	0.3 ± 0.1
15-epi-LXB_4	351	221	-	-	-
LTB_4	335	195	6.7 ± 1.0	5.6 ± 1.9	4.6 ± 1.8
PGD_2	351	189	21.4 ± 10.9	9.5 ± 1.9	11.6 ± 1.9
PGE_2	351	189	20.4 ± 4.8	11.1 ± 3.4	14.4 ± 3.3
$PGF_{2\alpha}$	353	193	8.0 ± 0.6	8.3 ± 1.7	7.8 ± 1.0
TxB_2	369	169	46.3 ± 9.3	56.8 ± 11.9	36.4 ± 10.7

Plasma was collected 2h after atorvastatin (0.2mg/Kg), pravastatin (0.2mg/Kg) or vehicle (DPBS containing 0.05% ethanol) administration on 7 day of inflammatory arthritis. Lipid mediators were identified in accordance with published criteria and quantified using multiple reaction monitoring of the parent ion (Q1) and characteristic daughter ion (Q3). Results are expressed as mean ± s.e.m.; *n* = 4 mice per group. - = below limits of detection; limits ~0.1 pg. Values denoted in bold are $P < 0.05$ *vs.* vehicle.

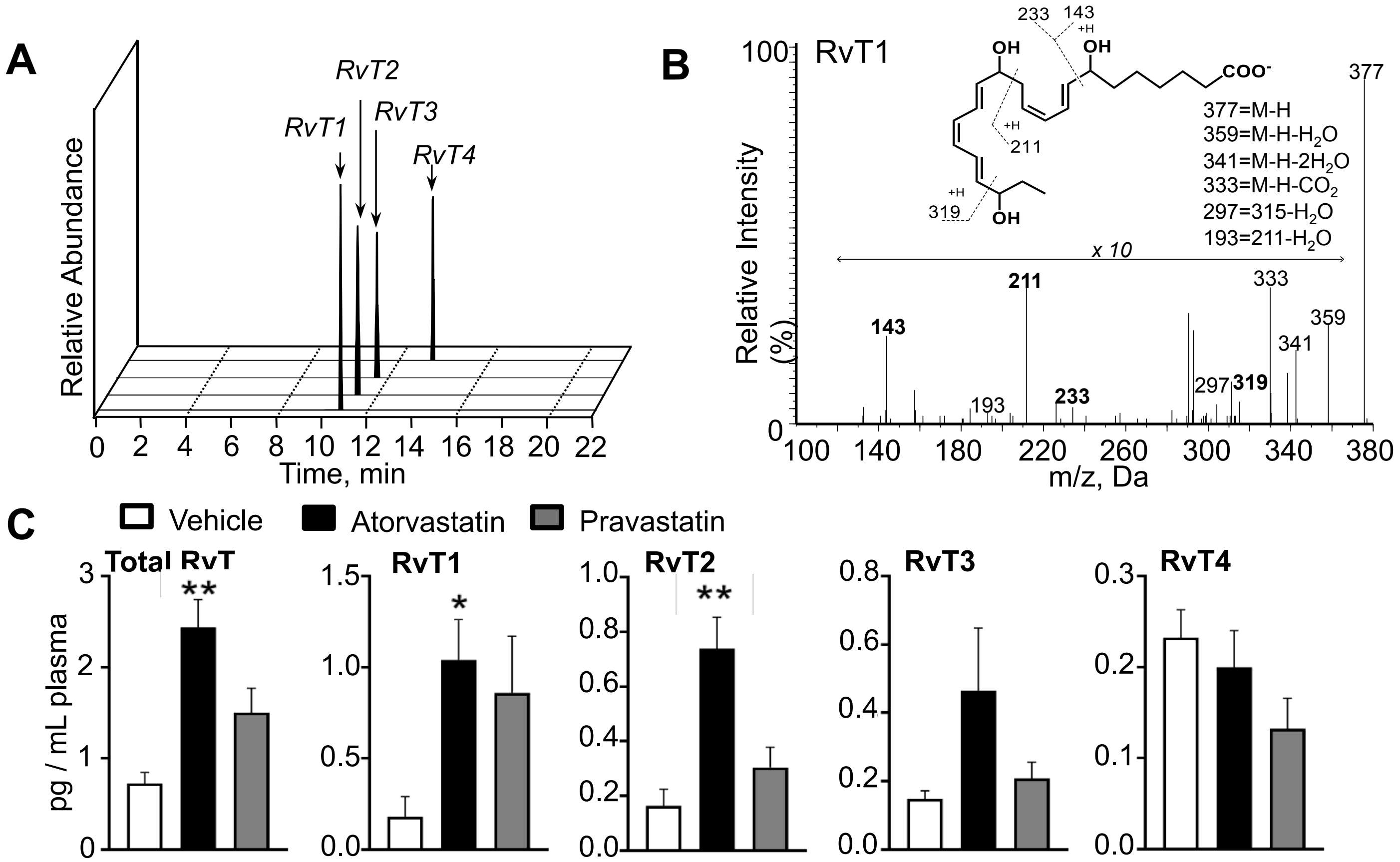

Supplemental Figure 1: Increased plasma RvTby atorvastatin and pravastatin during inflammatory arthritis. Serum-induced arthritis was initiated in mice and atorvastatin, pravastatin (0.2 mg/Kg each) or vehicle (DPBS containing 0.05% ethanol) was given on days 3, 5 and 7. Blood was collected 2 hours after statin administration on day 7 and RvT were identified and quantified using lipid mediator profiling. **(A)** Representative MRM chromatograms of identified $RvT_{n\text{-}3\ DPA}$. **(B)** MS/MS spectra employed in the identification of RvT1 (*inset*, diagnostic ions). **(C)** Quantification of total RvT, RvT1, RvT2, RvT3 and RvT4 compared to vehicle. Results for A, B are representative of n=12 mice, for C are mean ± s.e.m.; *n* = 4 mice per group. * $p < 0.05$ and ** $p < 0.01$ vs. vehicle using one-way ANOVA with *post hoc* Dunnett's multiple comparisons test.

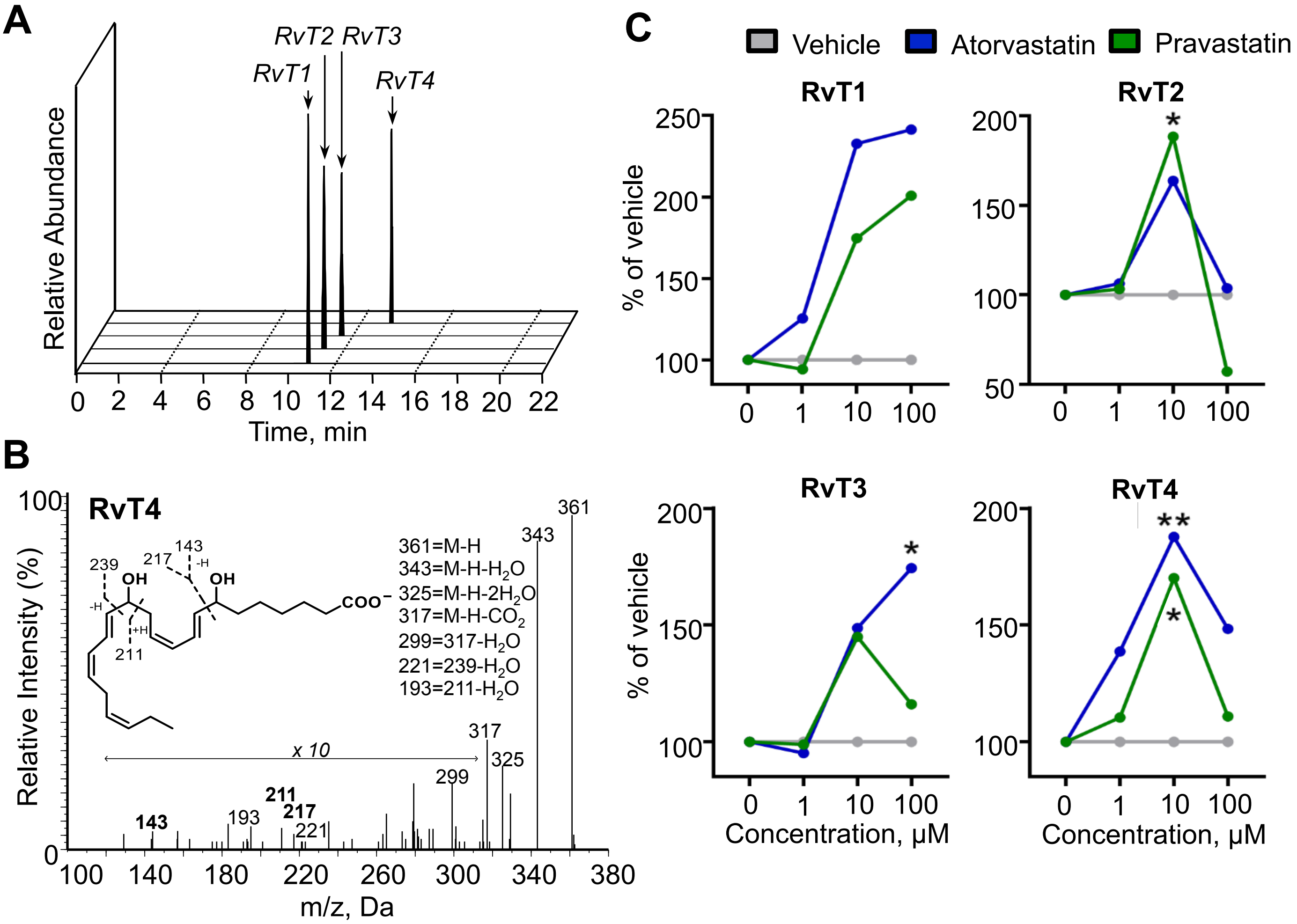

Supplemental Figure 2: Pravastatin dose-dependently increased RvT in human neutrophil-endothelial cell co-incubations. HUVEC (8.5 x 10^5 cells/cm^2) were incubated with IL-1β (10 ng/mL) and TNF-α (10 ng/mL) for 16h. These were then incubated with the indicated concentrations of atorvastatin, pravastatin or vehicle (DPBS containing 0.05% ethanol) for 30 minutes, then neutrophils (4 x 10^6 cells/well) were added. Incubations were quenched after 1h with 2 volumes of ice cold methanol and RvT were identified and quantified using lipid mediator profiling. **(A)** Representative MRM chromatograms of identified RvT. **(B)** MS-MS spectrum employed for the identification of RvT4. **(C)** RvT1, RvT2, RvT3 and RvT4 regulation compared with vehicle-treated incubations. Results are mean of 4 healthy donors from 4 independent experiments. * $p < 0.05$ and ** $p < 0.01$ vs. vehicle using two-way ANOVA with *post hoc* Tukey's multiple comparisons test.

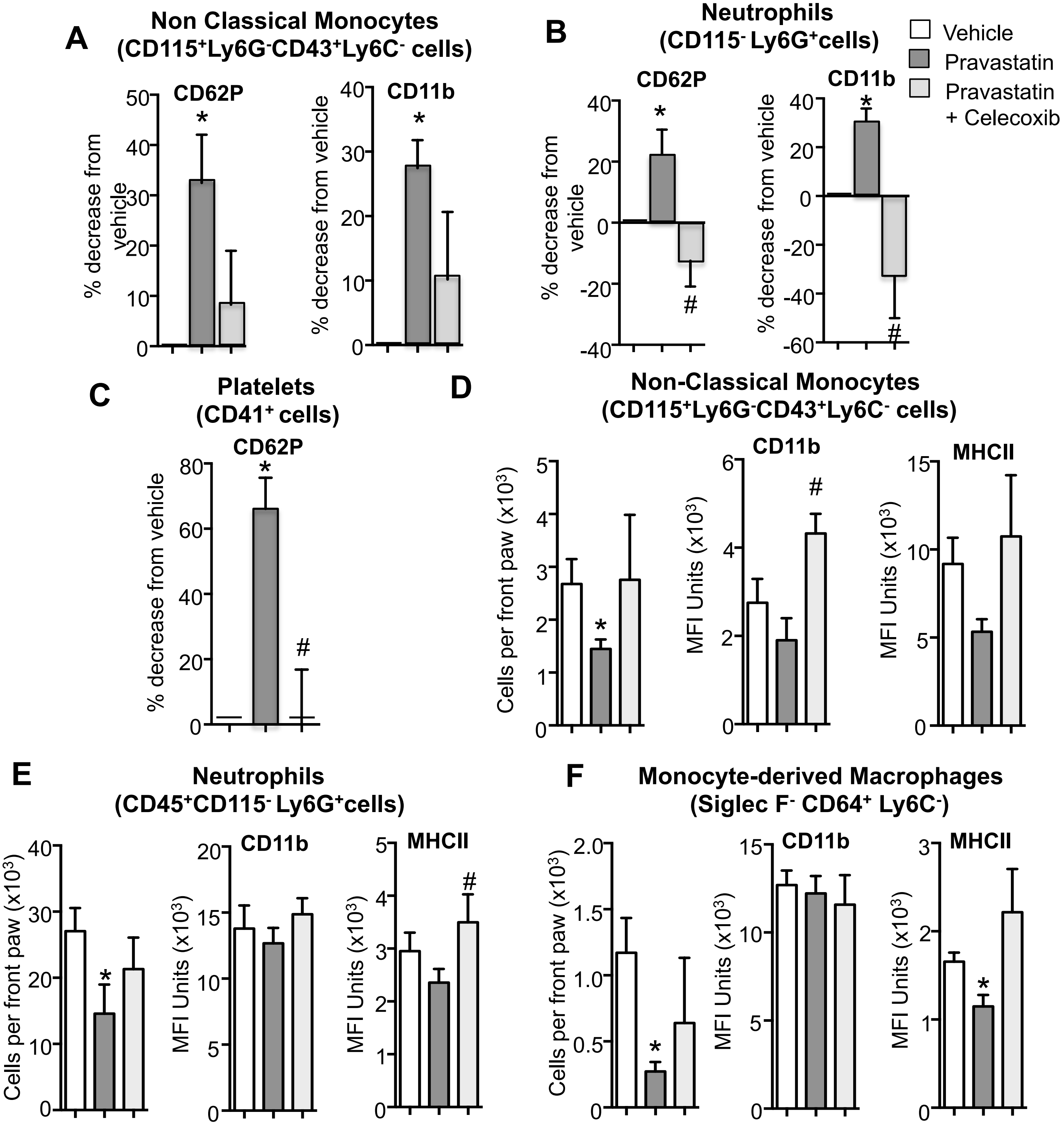

Supplemental Figure 3: Celecoxib reverses the protective actions of pravastatin on systemic and joint leukocytes. Serum-induced arthritis was initiated, ons days 3, 5 and 7 and mice were administered celecoxib (10 mg/Kg) or vehicle (DPBS containing 0.05% ethanol) and after 1 hour given pravastatin (0.2mg/Kg) or vehicle (DPBS containing 0.05% ethanol). Blood was collected on day 8 and leukocyte subsets and activation were identified using fluorescently labeled antibodies and flow cytometry. **(A-C)** Activation markers on circulating **(A)** non-classical monocytes, **(B)** neutrophils and **(C)** platelets. Results are presented as percentage decrease from vehicle. **(D-F)** Leukocytes recovered from the inflamed paws (see methods for details) on day 8. Trafficking and activation profile for (**D**) non-classical monocytes (**E**) neutrophils (**F**) monocyte-derived macrophages were assessed using flow cytometry. Results are mean ± s.e.m.; n = 9 for vehicle, 11 for pravastatin and 6 for celecoxib mice per group from 2 independent experiments. * $p < 0.05$ vs. vehicle using one-way ANOVA with *post hoc* Dunnett's multiple comparisons test.
